# Multidimensional eHealth Literacy for Infertility

**DOI:** 10.3390/ijerph17030966

**Published:** 2020-02-04

**Authors:** Susie Sykes, Jane Wills, Daniel Frings, Sarah Church, Kerry Wood

**Affiliations:** 1 School of Health and Social Care, London South Bank University, 101 Borough Road, London SE1 0AA, UK; 2 School of Applied Science, London South Bank University, 101 Borough Road, London SE1 0AA, UK

**Keywords:** digital health literacy, eHealth literacy, distributed health literacy, fertility health literacy, online fertility information

## Abstract

Infertility is a major public health issue and increasingly, the internet is used as a source of information and advice. The aim of this study is to understand the eHealth literacy of individuals and couples in relation to infertility. A non-probability sample of 27 participants was recruited from existing support groups, online advertising and snowballing representing the diverse population groups for whom involuntary childlessness is an issue. Information online was used both for decision making and developing interactive health literacy for health consultations. Participants may be both consumers and purveyors of information to others in distributed health literacy. Cognitive skills are required to appraise an inconsistent evidence base and potentially biased information from private providers of treatments. Accounts of geographical variations in treatment options, the cost of private treatment and for some, a sense that information and services were directed towards female and heterosexual couples, led some participants to political action online creating an important sense of empowerment. The study offers a new conceptual framework for eHealth literacy in the context of infertility, that combines use of the web and virtual communities in which functional, interactive, critical and distributed health literacy play a part in an online environment.

## 1. Introduction

Fertility is a key element of reproductive health, and infertility is recognized as a global public health issue by the World Health Organization (WHO) [1]. Infertility is defined by WHO as the ‘failure to achieve a pregnancy after 12 months or more of regular unprotected sexual intercourse’ [2] (p. 1522) and is experienced by an estimated 48.5 million couples worldwide [3]. The Internet is a common source of fertility-related information and over forty percent of women in the USA have reported using the internet as a primary source of information for infertility and resources [4]. Slauson-Blevin’s [5] analysis of 1352 women recruited from the US National Survey of Fertility Barriers (NSFB) was able to investigate different preferences to help-seeking by infertile women, demonstrating that online information was sought to complement rather than replace in-person information about infertility. The reasons for seeking information online may be to find out about causes of infertility, alternative treatments or ways to evaluate clinics [6,7]. Few studies have included other common help-seeking demographics (i.e., those thinking about future fertility planning, same-sex couples, or those who have had a live birth after a period of infertility). As many of these groups become more common with greater fertility planning, these groups are included in this study. 

Despite the recognized widespread use of the internet, relatively little is known about how individuals and couples use health portals and web-based information for infertility. Digital or eHealth literacy is the ability to seek, find, understand and appraise health information from electronic sources and apply the knowledge gained to addressing or solving a health problem [8]. Recent conceptualizations of eHealth literacy have identified seven domains that can be investigated: (1) How technology is used to process health information, (2) understanding of the health concepts and language in the content, (3) the individual’s ability to actively engage with digital services, (4) the individual’s feelings of being safe and in control when using online technology, (5) the individual’s motivation to engage with digital services, (6) access to digital services that work and (7) digital services that suit individual needs [9]. Making use of the internet demands both functional health literacy (the ability to access and understand information) and communicative or interactive health literacy (the personal skills needed to understand and act on information in a supportive environment). An early study [10] found that internet help-seeking could lead some with involuntary childlessness to withdraw from everyday interactions. In contrast, Hinton et al. [11] found that internet help-seeking reduced isolation, offering normalization and reassurance and was a social process involving shared resources and support which may be drawn from across a community as distributed health literacy [12,13]. Noorgard et al. [9] acknowledge that their eHealth Literacy Framework (eHLF) model does not fully incorporate these multi- dimensional characteristics of health literacy such as social support networks and engagement with professionals.

This study provides an opportunity to develop eHealth literacy frameworks based on the empirical findings that emerge from a study of online help-seeking in relation to the widely sought topic of infertility. The aim of this empirical investigation was to investigate whether (i) individuals used online information about fertility issues, (ii) how they engaged with it and (iii) whether it met their information needs. It was conducted by a team of five researchers working with health technology start-up companies using digital information. 

## 2. Materials and Methods

The study was interested in the experiences and perceptions of individuals and the meanings they attach to them. It therefore adopted an interpretive qualitative methodology allowing for the generation of the rich data and insight required to understand complex phenomena. It is reported following the consolidated criteria for reporting qualitative results (COREQ)guidelines [14]. Previous studies of online help-seeking have sought only the views of those who identify as involuntarily childless, but this study acknowledges that a much wider demographic group are interested in fertility information. A sampling strategy was therefore required that included individuals possessing characteristics relevant to meeting the research aim, rather than a random subgroup of the population. A non-probability purposive approach to sampling was therefore used to recruit participants of child-bearing age from five groups of interest: (1) people with infertility who have been trying to conceive for at least one year, (2) people who had a live-birth after a history of infertility, (3) people in same-sex relationships who are trying to conceive or want to explore their options for the future, (4) people who are not trying to conceive but want to learn about fertility and prepare for the future and (5) people who are aged 40 years or over who are trying to conceive. 

Participant selection took place through an invitation to participate which was posted through online fertility networks and social media sites including Twitter, Instagram and Facebook. Snowballing was employed as a secondary sampling strategy whereby referrals were taken from research participants for additional potential participants. Thirty-nine individuals responded to initial recruitment, of whom 27 agreed to be interviewed. Those not going on to be interviewed cited issues such as infertility as a stressful time, competing time pressures and work commitments. All participants were UK-based and were self-defined as of child-bearing age (women) or equivalent (men). Semi-structured telephone interviews were conducted with participants from each of the identified groups, as shown in Table 1. 

An interview schedule was developed based on the framework for eHealth literacy described by Norgaard et al. [9] to investigate for what purposes might the participant use the internet, how they engaged with technology and digital information, whether digital services met individual needs, their views about the trustworthiness and credibility of online information and services and how they used the information they found. The schedule was piloted with the first participant, but only minor amendments were made to the schedule as a result. Interviews lasted between 30 min and 1.5 h and were audio recorded with permission. Interviews were carried out by all five members of the research team. Checks were undertaken to ensure consistent use of the interview schedule through a team discussion following initial interviews and by the principal investigator conducting checks on audio recordings throughout the data collection period. 

Data were analyzed by two researchers using an inductive thematic analysis following the six steps laid out by Braun and Clarke [15]. NVivo (12) (QSR International, Melbourne, Australia) software was used to manage the analysis process. All transcripts were read by the first author for familiarization through the perspective of eHealth Literacy. The data were systematically coded followed by a process of inductively analyzing codes and their relationships to each other in order to generate meaningful subthemes and themes. Themes were mapped, reviewed and refined to ensure meaning and relevance. The first author led the analysis process with the second author reviewing each stage of the analysis. Both researchers were involved in the fifth stage of defining and naming the seven final themes. Ethical approval was secured for the project from the LSBU Health and Social Care Ethics Committee (ETH1819-0007). 

## 3. Results

Seven overlapping themes were constructed from the data that represent the participants’ experience of accessing fertility-related information online: the nature of the issue, a diversity of online users, motivations for accessing online information, accessing information, making sense of information and applying information for decision making and action. Each theme incorporates the differing facets from the data of the idea it represents and is made up of a number of related subthemes (see Table 2) illustrating the complexity and depth of the semantic data. 

### 3.1. Nature of the Issue—Stigma

The issue of fertility was perceived as particularly complex and surrounded by stigma or what some described as a ‘social taboo’, presenting challenges in discussing with friends and family or more widely in society.

*“It’s almost a stigmatized thing, so you sometimes find yourself at sea really.”*

(Participant 24)

For some, emotions of shame, denial and grief were expressed and experiences of isolation and an impact on relationships were expressed, all of which affected participants’ ability to discuss their situation openly:

*“I didn’t really talk to anyone, because I didn’t want to worry my mum, I didn’t want my friends to look badly of me. I felt really embarrassed and ashamed about being infertile-, talk to anyone.”*

(Participant 18)

These challenges were compounded by gender differences in how the issue is experienced:

*“I think my partner would struggle to understand exactly what I’ve been going through…. I can’t necessarily rely on friends or family because they don’t understand or know how to deal with it, some of them have, in fact, as soon as they’ve got pregnant stopped talking to me because I think they feel uncomfortable.”*

(Participant 24)

### 3.2. Diversity and Vulnerability

Participants described a wide range of personal circumstances, both of themselves and of those they met in online fertility forums, based on relationship status, gender, age, ethnicity, sexuality, socio-economic status and type of infertility being experienced. This diversity led to a wide range of need and experiences online, but some platforms were seen to assume a more homogenous audience. For some, vulnerabilities were identified and a potential for exploitation was felt particularly by those in same-sex relationships, some of whom felt removed and isolated from health services and any security they might offer:

*“Because you basically get straight men on there *[sperm donor websites]* that say, natural insemination only, which means having sex, and they basically think that lesbians are just going to have sex with them, like it’s really, like, predatory, you know, there’s a lot of like, just predatory men on there and so we, and again you have to pay the membership and stuff. Which I guess part of that is just so to like just filter out people misusing it, but it still happens.”*

(Participant 38)

Feelings of vulnerability and fear of financial exploitation were felt across the sample groups and were often linked to the complex nature of the fertility services and treatment, requiring navigation of a complicated private sector of competing services.

*“The industry isn’t very joined up. I went to the fertility show this year and I found that the medical professionals were very unprofessional…. They were almost bad mouthing each other and being quite competitive about things. It made me feel very uncomfortable.”*

(Participant 10)

### 3.3. Motivations for Accessing eHealth Information

Participants were motivated by two key factors when seeking information online: a pursuit of information about fertility and the services available to them and secondly, stories about personal experiences. While a minority did not want to engage with people’s personal experiences, the majority did. For the latter, personal stories were seen as an important source of information, providing a more holistic and human angle and enabled participants to find somebody whose situation reflected their own:

*“I think to me personally, it was good to see personal stories on those websites, and particularly about miscarriages. There were a lot of personal stories and how people cope with this and what they do next. So, that was definitely helpful to me and I could see that natural human beings interact with this website, with this group. To me, that was helpful.”*

(Participant 16)

### 3.4. Accessing Information Online: Where to Go and What to Look For

Accessing online information was described as a complex process and issues identified included: first point of access online, types of platforms accessed, topics searched and gaps in information.

All participants said that they accessed the internet as one of their first sources of information. Frustrations were expressed at the limited information provided by health professionals, especially when first contacting them about fertility issues:

*“Just to share my example of reaching out to doctors in the NHS, they were completely uninterested and didn’t really want to have the conversation, didn’t want to give me the blood test, didn’t offer me any support or advice. …They didn’t offer me any further reading or research, or anything like that.”*

(Participant 11)

The two motivating factors identified above result in individuals accessing both Web 1.0 platforms (traditional, information-based websites, where users view content in a passive manner) and Web 2.0 platforms (websites that allow user collaboration and includes social networking sites, social media sites, blogs, wikis and video sharing sites). Those searching for the former inevitably began their search in a web browser, typically Google, and searching was generally described as unstructured and relied on some luck to find useful sites:

*“Really, I suppose it is a bit of a scattergun approach. It’s seeing what comes up on Google.”*

(Participant 17)

Web 2.0 platforms included message and discussion boards but these were often seen as out of date and containing historic content that was frustrating to search. Social media sites were more frequently mentioned including Facebook, Twitter and Instagram. Instagram was by far the most popular social media platform with participants suggesting they attracted different audiences and reporting a more informed conversation on Instagram than on Facebook:

*“I think the people who like Instagram and use Instagram are very different from the people that would use a forum or would use Facebook. I know pretty much, I would say, 90% of the women that I network with online, they absolutely hate Facebook and they wouldn’t use it on a personal level, or even a professional level. They don’t like it. I think it’s probably to do with our generation, it’s probably to do with the way that people overshare information.”*

(Participant 10)

Information sought varied as participants progressed through their journey to infertility treatment or not. Typically, very general searches were undertaken initially, often related to causes of infertility, and most felt this level of information was generally well provided:

*“Initially it was just general information, like ’we can’t conceive, what do we do now?’ or ’how soon do we go to the doctor?’ ’what percentage of couples conceive before their treatment starts, so before the year’s up or the two year’s up.”*

(Participant 20)

As individuals became more informed and engaged with services and treatment more fully, the topics they looked for became more bespoke and more challenging to find.

*“I think there’s a lot to get people started. I think there’s a lot. I think it’s probably when you get to a certain stage and it could be a very, very specific medical, genetic reason why, say, your cycles keep failing or you keep miscarrying. That’s probably where it gets tricky and you might not find information online.”*

(Participant 9)

Many were also looking for emotional support and a sense of community. Those using the social media platforms did use them for information but many also used them to find people in similar situations and form support networks which, for some, became very close and evolved into offline friendships. Support online rather than from real world friends and family was preferred by some because of the shared experience and ability to discuss openly and anonymously:

*“In my situation, I became part of a group on Facebook that is for women that are trying to have a baby alone. … There’s nearly 500 of us on there. That has been a huge source of information to me, huge, because obviously all these women are in the same situation… I’ve become very, very good friends with probably about six women there that have had children now, who have got very small babies, and I go and help them all the time.”*

(Participant 3)

The most frequently cited information gap was male factor infertility. It was also felt that content online did not sufficiently reflect the diversity of people that might be seeking information on fertility, particularly those not in relationships or in same-sex relationships. These latter participants felt that information on topics they might be most interested in was harder to find and at times, appeared less legitimate:

*“When you’re looking at home insemination I think sometimes it felt a bit like more taboo, like, it’s more off-the-book if that makes sense. Like, you’re not really supposed to be doing this, but actually when you get the legal information it’s like all of this is legal, it is okay, we’re not doing anything, like, suspicious we’re not like, paying for a baby and stuff like that.”*

(Participant 38)

### 3.5. Navigating Sites—How to Manage It

Once participants engaged with a site, they faced challenges including managing the information and the style in which information was presented. Many participants felt overwhelmed by the information available and there was also a sense that the basic and general information that might be useful is available on many sites but that quickly becomes redundant as more specific and specialized information is required.

The aesthetic of a site was seen as very important with a preference expressed for simple, clean, uncluttered and professional sites. Adverts were seen very negatively as were sites that were too impersonal and made use of chat-bots:

*“The little chat-bot, the AI thing pops up and wants to chat, kind of, like, well, this isn’t something that you want to chat to a computer about.”*

(Participant 11)

While professionalism was important, so too was a personal element and a sense of who is behind the website and their motivation. A challenging balance of non-medicalized language that was not over simplified or patronizing was desired:

*“I don’t know if other people feel the same as me but I sometimes find websites patronizing as they go on too much about how you should try and be healthy, so, I would be a bit, kind of, careful you know, assuming that people who have tried for years, they probably know they should eat their vegetables.”*

(Participant 2)

For some, optimism was appreciated, but for most, a realistic tone was more important. Participants wanted infertility to be presented in a way that normalized it and helped remove the perceived taboo. There was also a desire for information to be presented in a more inclusive way as there was a feeling that most sites targeted white, heterosexual females in relationships and that this did not reflect reality. This was seen to be apparent in images, language and content:

*“It would be nice if they consider, like, to try and appeal to men and women because I know for my husband, there’s a lot less out there and also for different problems because a lot of websites are about female infertility issues and a lot of the support online is for those.”*

(Participant 2)

### 3.6. Making Sense of Information

In discussing how information was appraised and processed, two themes emerged: the issue of trustworthiness and a process of collective analysis and meaning making of information described in this section as distributed health literacy.

The trustworthiness of information was an important issue for participants. Individuals felt that infertility is a rapidly changing field and there is an inconsistent evidence base. Infertility treatment is often sought from private providers and so information online can be biased and was often contradictory:

*“Some people will say, actually, Tupperware is the devil and you should not have any BPA, but actually, other people say you can’t really avoid it, and it’s about minimizing it as much as you can. It’s not really clear, actually, what does the evidence say?”*

(Participant 17)

Some participants were also aware of their own bias and that their desperation to find certain answers might lead them to keep searching until they found them, making them vulnerable to misinformation:

*“It’s more me wanting to find something rather than just me dismissing something because it seems like hearsay or gossip. I’m not able to withdraw at that stage.”*

(Participant 24)

In judging trustworthiness, a credible source was very important. Participants were keen to see a site where a trusted organization sifted through and appraised evidence for them and translated it into plain English:

*“That’s why if I find a site that, sort of, collects all this data for me, in a way, and, sort of, screens them for me, that would make it helpful. That would make the work a bit easier and present the information a bit more systematically”.*

(Participant 29)

There were conflicting opinions over whether information based on personal information was more or less trustworthy. For some, personal opinion was to be avoided because it was not fact-based:

*“Just because it’s not based on facts. It’s people-, experiences and not necessarily backed up information that they’re sharing.”*

(Participant 12)

While for others, the opposite was true and the personal nature of it made it more reliable:

*“You know, because some people share their personal experiences of it. For me, it’s hard, just to doubt that.”*

(Participant 29)

Others recognized this conflict in themselves:

*“So, I guess the trust element as well. I would trust a scientific journal article over, maybe, a blog that someone’s written, but then a blog is also personal experience, so actually, that statement might not be true. It’s more real then, isn’t it? It’s more tried and tested.”*

(Participant 9)

As part of the process of finding, appraising and processing information, participants moved backwards and forwards continuously between the Web 1.0 and Web 2.0 platforms. Online communities were proactively used to identify sources of information and then to discuss, process and understand information. Through this, an important collective understanding was generated. People engaged in this process differently with some passively observing conversations while others engaged in conversations or led them:

*“I think I feel it’s a very safe place for information, and I wouldn’t have known about DNA fragmentation unless I had joined this community, because there were other women who were desperate to get help and found out more about why their IVF was failing, and to see specialists because their husbands hadn’t been given the time of day. Then they share who they’d seen, what investigations they’ve had done, and then you’ve got that in your mind and you think, ’Do you know what? I’m going to ask about this, and I’m going to look it up and I’m going to find out more information.”*

(Participant 10)

### 3.7. Using Information for Decision Making and Action

In using the information gathered online, a number of themes emerged. Information was used for decision making as well as for preparing for, and managing, consultations, described here as interactive health literacy. A movement was also described from participants as consumers of information to purveyors of information and ultimately, for some, demonstrating levels of critical health literacy skills. 

Information gathered from the internet and discussed online was consistently used as the basis for action. Information gained informed decision making and underpinned changes in behavior:

*“So, that made me more aware of what I was eating, what I was drinking, what tablets I was taking, nutrition, all of that kind of stuff, so I googled probably a lot about that as well then, and acupuncture. You know, like, if I eat pineapple, will that help? That kind of stuff, Brazilian nuts, and you know, the fertility diet type of thing.”*

For most participants, information was used to inform decision making about treatment options:

*“I looked at the NICE guidelines, which says that at the moment there is no evidence to suggest that that would improve rates, that particular surgery, so that is not advised. So, that’s why I talked about it with my husband. I would go there’s the evidence and say if there’s no evidence you shouldn’t do it, it’s still surgery, there’s still risks attached to it.”*

(Participant 2)

Participants frequently cited examples of using information from websites and online conversations to prepare for, and to understand, interactions with health professionals. Participants used information and other people’s experiences to prepare questions for health professionals and to ask about treatment options, ensuring a more informed conversation. They then took information from their consultation back to the online communities in order to assess options and develop their understanding. This iterative process was highly valued:

*“I just wanted to know what to expect, rather than just walking into the surgery or walking into hospital not really knowing. Even just little things, like, I didn’t know what to wear, and you know, whether I needed to-, just being prepared to have to take my clothes off, or to have an examination or something like that. Just so I felt a bit more prepared before going to the doctors, really.”*

(Participant 12)

Many talked about how quickly their knowledge had grown and how they had become ‘experts’ in the subject describing a transition from being consumers of knowledge to becoming purveyors of knowledge, initially to their partner or family, but for some, this progressed to other users in the online community. For some it went further and they set up their own sites, wrote their own blogs or contributed articles to existing sites providing an important sense of purpose.

*“Now, I’ve written a couple of articles and stuff like that have been published, not for payment, just on some of those websites.”*

(Participant 23)

## 4. Discussion

The example of infertility illustrates the important function the internet plays as a source of both information and support. The taboo identified in owning and discussing infertility in public spheres, has been evidenced elsewhere [16,17] and this places a premium on digital platforms for information. The gendered pursuit and provision of fertility information [7,17] and the concerns that online fertility platforms do not recognize the diversity of personal circumstances leads to a danger that this important resource is not equally accessible to all. This is compounded by the complex eHealth literacy skills required to navigate this information. Those with higher levels of eHealth literacy have been shown to gain more positive outcomes from internet use in terms of knowledge, behavior and interactions with health professionals [18,19], and this leads to concerns that inequalities already experienced with regard to infertility may become compounded.

Individuals are motivated to access the resources held online for a number of purposes: health information, service information, the appraisal of information against knowledge and experience, preparing for and understanding consultations, help in decision making and emotional support. How people engage with information and behave within online communities varies, with some taking a passive role as observers and consumers of information, others actively engaging in discussion and support and some taking a facilitating or leadership role. The accessing of online resources has the potential to lead to a more informed and supported individual who can become a purveyor of information and engage in informed decision making and action.

**Figure 1:**
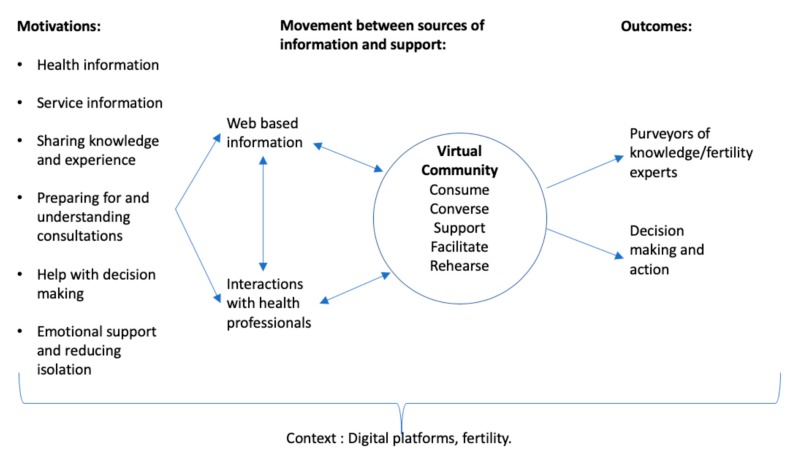
New conceptualization of eHealth Literacy in context of infertility.

eHealth literacy models have been helpful in understanding how people interact with digital platforms and the skills they need to access, understand, appraise and apply health information. The original model of eHealth Literacy offered by Norman and Skinner [8] has been used as the conceptual foundation for work in this area. Norman himself identified the need for his Lily model of eHealth literacy to be further developed in light of digital developments, in particular the context of Web 2.0 opportunities. However, subsequent conceptual developments, for example in work by Chan and Kauffman [20], Gilsted [21] and more recently by Noorgarrd et al. [9], still focus on the interaction of users with technology and digital services rather than the two-way dialogical nature of online opportunities offered through social media platforms. Work by Chen and Lee [22] demonstrated the importance of participatory eHealth behaviors alongside the traditional focus on informational eHealth behaviors. What the current study adds to this discussion is a demonstration of the complex interaction and movement by participants between: information-based platforms, conversational platforms and information provided by health professionals with online communities playing a central part in this relationship. The findings demonstrate that this movement contributes to all stages of accessing, understanding, appraising and applying information. Importantly, this suggests that the gap in conceptualizations of eHealth Literacy identified by Norman [8] has not yet been addressed. Reflecting this and our findings, Figure 1 is a new conceptual model of eHealth literacy that shows these complex and diverse ways in which people use the internet as a resource for health literacy in the context of fertility. 

Existing work in this area has shown the importance of established networks to access information and support decision making [23,24] and this study demonstrates the value that people seeking infertility information place on such networks. Where physical networks are absent, these findings show an active pursuit to join them or create them in an online environment and may be understood as an example of distributed health literacy. This concept suggests that the knowledge and skills required to be health-literate are not just held by an individual but are an available resource distributed across a whole community [25]. This shared resource can act as a buffer for people with low levels of health literacy [25,26], with one study showing that each percentage increase of average health literacy within a community is associated with a two percent increase in self-reported health for individuals in that community, concluding that both individual- and community-level health literacy are significant, distinct correlates of individual health status [13]. McElhinney’s work [27] on Virtual World communities, while focusing on avatar-based immersive environments, has shown how the collective knowledge and skills of communities in Virtual Worlds can influence and improve individual health literacy in the physical world. The use of the online environment as a safe space to prepare for consultations with professionals and therefore build interactive health literacy, may offer important potential, with McElhinney’s work [28] illustrating how the rehearsing of learning in a virtual world can act as a pre-curser to the practicing of it in the physical world. However, how distributed health literacy is operationalized in an online environment and its impact on individual decision making and action is still poorly understood. 

While this example of distributed health literacy offers the sharing of knowledge and skills across a network, it also offers the potential for the spread of misinformation [29] and the supporting of negative decision making, as demonstrated by McKinn et al. [26]. The trustworthiness of information about fertility was important to the participants but there were considerable challenges associated with judging credibility, the availability of conflicting information and their own emotional drivers that led some participants to actively seek a particular conclusion. These challenges exist not only in distributed health literacy in online communities but also for those relying on web-based information where discrepancies between internet and academic literature exist [30,31]. Calls for the participatory development of digitally based health information [32] may go some way to mitigate against the latter, but this is harder to address within a social media context. The Online Health Community model presented by Zhou and Fan [33] of health communities managed by healthcare providers or expert patients may offer some potential, and these are increasingly seen to be forming a component part of targeted health apps. The HealthUnlocked app offered by the NHS in the UK offers 700 online communities moderated by patient organizations. However, their acceptability and use as an alternative to naturally formed virtual communities is not well explored, particularly within the field of fertility.

A limitation of this study is its geographical focus. All participants were from the UK and focused on the use of English language internet sites. The cultural and contextual issues raised and those relating to the nature of service provision may therefore vary in other geographical settings. The sampling strategy also utilized infertility networks as a key forum for recruitment. This may have meant that those very early on in their pursuit of infertility information may have been less likely to have been included in the sample. Finally, sampling purposively focused on people with specific experiences (e.g., a diagnosis of infertility, having had a live-birth after a history of infertility) rather than age, ethnicity or socio-economic factors. This was to ensure a recruitment priority was placed on the identified groups experiencing infertility. Variations may exist in the experiences across child-bearing age, ethnicity and socio-economic status that are not captured by this study. While these data may not be generalizable to the whole population experiencing infertility, the recurrent appearance of themes and absence of new codes being generated towards the end of the analysis process, as well as the similar sample size to other studies in this area [11,34], means that data adequacy can be seen to have been achieved. 

## 5. Conclusions

A new conceptualization of eHealth literacy was developed from this study that more fully acknowledges the importance of online communities and the role that distributed health literacy may play in an online environment. The continual movement between the digital spaces of web-based information and virtual communities, and the physical world interactions with health professionals is an important finding for eHealth literacy models. It has important implications for information providers in showing how online information has the potential to contribute to functional, interactive and critical health literacy. 

The use of digital platforms is affected by the nature and topic of the health information sought. Whilst studies of online help-seeking use a range of theoretical explanatory models [35], predominantly informed by psychological expectancy about what needs information will serve, individual motivations in relation to infertility are varied and complex, as shown in Figure 1. As a sensitive health issue surrounded by stigma and where there is a varied demographic and a diversity of circumstances and needs, online help-seeking for infertility illuminates the importance of communities and the transactional nature of the relationship that the individual user has with the digital information.

## Figures and Tables

**Table 1:** Participants.

Sample Group	Female	Male	Total
Individuals with infertility who have been trying to conceive for at least 1 year	14	0	14
Individuals who had a live-birth after a history of infertility	7	1	8
Individuals in same sex relationships who are trying to conceive or want to explore their options for the future	2	1	3
Individuals who are not trying to conceive but want to learn about fertility and prepare for the future	2	1	3
Individuals who are aged 40 or over who are trying to conceive	3	0	3
Total Sample	24	3	27

Note: Each participant may appear more than once if they identified with multiple groups.

**Table 2:** Data themes and corresponding subthemes.

Theme	Subtheme
1. Nature of the issue	Stigma
	Inability to discuss
	Impact on relationships
	Emotional impact
	Isolation
	Gender differences
2. Diversity and vulnerability	Relationship status
	Sexuality
	Gender
	Fertility related issue
	Economic circumstances
	Financial vulnerability
	Complexity of services
	Private sector nature of services
3. Motivations for accessing eHealth information	Pursuit of information about fertility issues
	Pursuit of information about services
	Pursuit of information about personal stories
4. Accessing information online	First point of access
	Accessing information-based websites
	Accessing collaborative and social networking sites
	Fertility topics searched
	Emotional support and support networks
	Information gaps
5. Navigating sites	Managing information
	Style of information
	Appropriate detail information
	Aesthetic
	Professionalism
	Language
	Optimism/realism
	Inclusiveness
6. Making sense of information	Trustworthiness
	Internal bias
	Collective analysis and meaning making
7. Using information for decision making and action	Information for decision making
	Information for preparation for consultations
	Experts and purveyors of information
